# Defective T-cell control of Epstein–Barr virus infection in multiple sclerosis

**DOI:** 10.1038/cti.2016.87

**Published:** 2017-01-20

**Authors:** Michael P Pender, Peter A Csurhes, Jacqueline M Burrows, Scott R Burrows

**Affiliations:** 1 School of Medicine, The University of Queensland, Brisbane, Queensland, Australia; 2 Department of Neurology, Royal Brisbane and Women’s Hospital, Brisbane, Queensland, Australia; 3 Cellular ImmunoIogy Laboratory, QIMR Berghofer Medical Research Institute, Brisbane, Queensland, Australia; 4 The University of Queensland Centre for Clinical Research, Brisbane, Queensland, Australia

## Abstract

Mounting evidence indicates that infection with Epstein–Barr virus (EBV) has a major role in the pathogenesis of multiple sclerosis (MS). Defective elimination of EBV-infected B cells by CD8^+^ T cells might cause MS by allowing EBV-infected autoreactive B cells to accumulate in the brain. Here we undertake a comprehensive analysis of the T-cell response to EBV in MS, using flow cytometry and intracellular IFN-γ staining to measure T-cell responses to EBV-infected autologous lymphoblastoid cell lines and pools of human leukocyte antigen (HLA)-class-I-restricted peptides from EBV lytic or latent proteins and cytomegalovirus (CMV), in 95 patients and 56 EBV-seropositive healthy subjects. In 20 HLA-A2^+^ healthy subjects and 20 HLA-A2^+^ patients we also analysed CD8^+^ T cells specific for individual peptides, measured by binding to HLA-peptide complexes and production of IFN-γ, TNF-α and IL-2. We found a decreased CD8^+^ T-cell response to EBV lytic, but not CMV lytic, antigens at the onset of MS and at all subsequent disease stages. CD8^+^ T cells directed against EBV latent antigens were increased but had reduced cytokine polyfunctionality indicating T-cell exhaustion. During attacks the EBV-specific CD4^+^ and CD8^+^ T-cell populations expanded, with increased functionality of latent-specific CD8^+^ T cells. With increasing disease duration, EBV-specific CD4^+^ and CD8^+^ T cells progressively declined, consistent with T-cell exhaustion. The anti-EBNA1 IgG titre correlated inversely with the EBV-specific CD8^+^ T-cell frequency. We postulate that defective CD8^+^ T-cell control of EBV reactivation leads to an expanded population of latently infected cells, including autoreactive B cells.

Mounting evidence indicates that infection with the Epstein–Barr virus (EBV) is a prerequisite for the development of multiple sclerosis (MS), although its exact role is incompletely understood.^1, 2^ EBV, a ubiquitous double-stranded DNA γ-herpesvirus, is unique among human viruses in having the capability of infecting, activating, clonally expanding and persisting latently in B lymphocytes for the lifetime of the infected person. To accomplish this, EBV utilizes the normal pathways of B-cell differentiation.^3^ During primary infection EBV is transmitted through saliva to the tonsil where it infects naive B cells and drives them out of the resting state into activated B blasts, which then progress through a germinal centre reaction to become circulating latently infected memory B cells.^3^ When latently infected memory B cells returning to the tonsil differentiate into plasma cells, the infection is reactivated by initiation of the lytic phase culminating in the generation of virions,^4^ which infect tonsil epithelial cells where the virus reproduces at a high rate and is released into saliva continuously for transmission to new hosts.^5^ Newly formed virus also infects additional naive B cells in the same host, thereby completing the cycle necessary for its persistence as a lifelong infection.^6^ To pass through the various stages of its life cycle, EBV makes use of a series of differing transcription programmes.^3^ After entering naive B cells, it first employs the latency III or ‘growth’ programme expressing all viral latent proteins, namely the Epstein–Barr nuclear antigens (EBNA) 1, 2, 3A, 3B, 3C and LP, and the latent membrane proteins (LMP) 1, 2A and 2B, to activate the blast phase. After entering a germinal centre, the infected blast switches off expression of the EBNA proteins 2, 3A, 3B, 3C and LP and continues to express EBNA1, LMP1 and LMP2 (latency II or ‘default’ programme) while it progresses through the germinal centre phase to differentiate into a memory B cell. Because latently infected memory B cells express no viral proteins they are unable to be detected by EBV-specific immune responses, except during cell mitosis, when they express only EBNA1 (latency I), which is needed for duplication of the EBV genome and transmission to daughter cells. When latently infected memory B cells differentiate into plasma cells the virus is reactivated through the lytic transcription programme to generate infectious virions.

In healthy individuals, EBV infection is kept under rigorous control by EBV-specific immune responses, especially by cytotoxic CD8^+^ T cells, which kill proliferating and lytically infected B cells by targeting the various EBV-encoded latent and lytic proteins respectively.^7, 8^ We have hypothesized that defective elimination of EBV-infected B cells by cytotoxic CD8^+^ T cells might predispose to the development of MS by enabling the accumulation of EBV-infected autoreactive B cells in the central nervous system (CNS).^9, 10^

On the basis of expression of CD45RA, CCR7 and CD62L, human CD4^+^ T cells and CD8^+^ T cells can be divided into four major subsets with different homing and functional properties, namely: naive (CD45RA^+^CCR7^+^CD62L^+^); central memory (CM) (CD45RA^−^CCR7^+^CD62L^+^); effector memory (EM) (CD45RA^−^CCR7^−^CD62L^−^); and effector memory re-expressing CD45RA (EMRA) (CD45RA^+^CCR7^−^CD62L^−^) cells.^11, 12^ Naive and CM CD8^+^ T cells home to secondary lymphoid organs, whereas EM and EMRA CD8^+^ T cells travel to inflamed non-lymphoid tissues and have effector functions such as IFN-γ production and cytotoxicity. In MS there is a general deficiency of CD8^+^ T cells predominantly involving the CD62L^−^ effector memory (EM/EMRA) subset,^13^ which carry out immunosurveillance of the CNS and protect against viral infection.^14, 15^

Studies investigating T-cell immunity to EBV in MS have yielded conflicting results, with reports of decreased,^16, 17, 18, 19, 20, 21^ normal^22^ and increased responses.^23, 24, 25, 26, 27^ These discrepancies are likely to be largely due to differences in the EBV antigens, T-cell populations, T-cell functions and stages of MS studied.^2^ To address this important issue we have undertaken a comprehensive analysis of the T-cell response to EBV in MS, incorporating the following features: (i) commencing with measurement of the total T-cell response to the physiologically relevant target, namely EBV-infected B cells, then assessing the response to pools of human leukocyte antigen (HLA)-class-I-restricted peptides from EBV lytic or latent proteins, and from another herpesvirus, cytomegalovirus (CMV), and finally measuring the response to individual EBV lytic and latent peptides; (ii) analysis of the responses of both the total CD4^+^ T-cell and CD8^+^ T-cell populations and also their memory subsets; (iii) analysis of both the phenotype and function of EBV-specific CD8^+^ T cells using peptide-HLA-class I multimers and intracellular cytokine staining to measure the secretion of IL-2, TNF-α and IFN-γ (iv) correlation of the cellular immune response to EBV with the humoral immune response and the EBV DNA load; (v) analysis at all stages of the disease process, from onset to late in the progressive phase; and (vi) correlation of immune responses to EBV with the clinical course of MS. We found that at all stages of MS there is a decreased CD8^+^ T-cell response to lytic phase EBV, but not lytic phase CMV, antigens indicating impaired control of EBV reactivation. In contrast CD8^+^ T cells directed against EBV latent antigens are increased in number, but reduced in function, indicating an exhausted response to the expanded population of latently infected cells resulting from reduced CD8^+^ T-cell control of EBV reactivation.

## Results

### The T-cell response to EBV-infected B cells is reduced at all stages of MS except during clinical attacks

The demographic and clinical details of the healthy subjects and patients with MS are presented in Table 1. The patients with MS had a decreased frequency of total CD8^+^ T cells (Supplementary Table S1) and, in particular, total CD8^+^ EM/EMRA T cells (Supplementary Table S2), as we have previously reported.^13^ We first measured the total T-cell response to the physiologically relevant target, namely EBV-infected B cells, using flow cytometry and intracellular cytokine staining to measure the frequencies of T cells within peripheral blood mononuclear cells (PBMC) producing IFN-γ in response to autologous EBV-infected lymphoblastoid cell lines (LCL) in 56 EBV-seropositive healthy subjects and 95 patients with MS. LCL express not only the latent proteins of EBV but also the lytic proteins,^28, 29^ owing to the fact that a proportion of the cells in LCL are in the lytic phase of infection. This approach provides a direct measure of the aggregate T-cell response to EBV-infected B cells in each subject because it uses each person’s natural antigen-processing mechanisms to present viral antigens at normal physiological concentrations on the surface of their own EBV-infected B cells and it represents the total T-cell response to all EBV antigens presented by all HLA molecules on infected B cells in each subject.^19^ The specificity of the T-cell response to EBV using this approach was demonstrated by the fact that the median frequencies of LCL-reactive CD4^+^ T cells and CD8^+^ T cells in PBMC were 0.000% and 0.000%, respectively, in six EBV-seronegative healthy subjects, compared with 0.035 and 0.183% respectively in 56 EBV-seropositive healthy subjects (*P*<0.0001 for both comparisons; Supplementary Table S3). Because a difference in the proportion of lytically infected cells within LCL between MS patients and healthy controls could lead to a difference in the ability of LCL stimulation to detect EBV-lytic-specific T cells, we measured expression of the EBV immediate early lytic protein BZLF1 in LCL of 19 healthy EBV-seropositive subjects and 56 patients with MS at the same time as the measurement of the LCL-specific T-cell frequency. The median frequency of BZLF1^+^ cells within LCL was 2.15% in healthy subjects and 2.02% in the patients (*P=*0.979), indicating that there was no difference in the proportion of lytically infected cells within LCL between MS patients and healthy controls.

**Figure 1:**
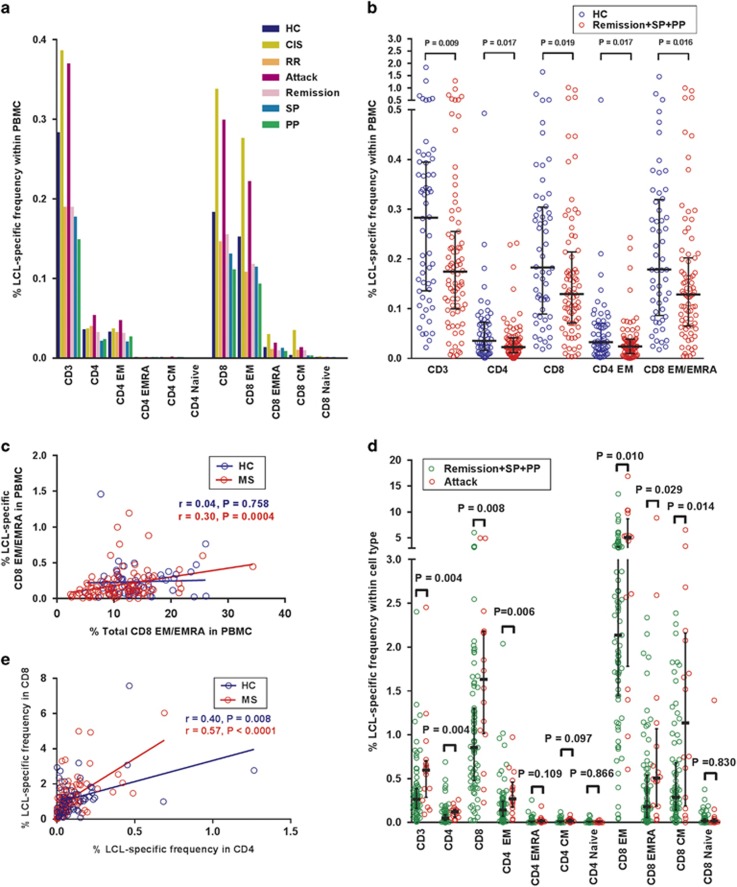
Frequencies of T cells producing IFN-γ in response to autologous EBV-infected LCL. (**a**) Median percentages of T cells of different phenotypes producing IFN-γ in response to autologous EBV-infected LCL in the PBMC in EBV-seropositive healthy controls (HC), patients with clinically isolated syndrome (CIS), patients with relapsing–remitting (RR), secondary progressive (SP) and primary progressive (PP) MS, as well as patients during a clinical attack (Attack) and during remission (Remission). (**b**) Percentages of LCL-specific T cells in the PBMC in patients not having a clinical attack (Remission+SP+PP) compared with HC (medians with interquartile ranges indicated by black horizontal lines), with bracketed *P* values determined by the Mann–Whitney test. (**c**) Percentage of LCL-specific CD8^+^ EM/EMRA T cells in the PBMC plotted against the percentage of total CD8^+^ EM/EMRA T cells in the PBMC in healthy subjects (HC) and the total group of MS patients (MS). (**d**) Percentages of LCL-specific cells within the CD3^+^, CD4^+^, CD8^+^, CD4^+^ EM, CD4^+^ EMRA^+^, CD4^+^ CM, CD4^+^ naive, CD8^+^ EM, CD8^+^ EMRA, CD8^+^ CM and CD8^+^ naive T-cell populations in patients during a clinical attack (Attack) compared with patients not having an attack (Remission+SP+PP) (medians with interquartile ranges indicated by black horizontal lines), with bracketed *P* values determined by the Mann–Whitney test. (**e**) The frequency of LCL-specific T cells within the CD8^+^ population strongly correlated with the frequency of LCL-specific T cells within the CD4^+^ population in MS patients whereas this correlation was weaker in healthy subjects. On multiple linear regression analysis, the slope of the regression line in the MS patients was significantly greater than that in healthy subjects (*P=*0.006).

In healthy EBV-seropositive subjects the CD8^+^ T-cell response to LCL was much greater than the CD4^+^ T-cell response, as we have previously reported.^30^ Analysis of T-cell memory phenotype based on CD45RA and CD62L expression revealed that LCL-reactive CD4^+^ T cells were predominantly of the EM type and LCL-reactive CD8^+^ T cells were predominantly of the EM type and less frequently of the EMRA type (Figure 1a). We obtained similar results when CCR7 was used instead of CD62L to analyse memory phenotype. For simplicity, all the analyses of memory phenotype presented in this study are based on the expression of CD45RA and CD62L. The frequencies of LCL-specific T cells were decreased at all stages of MS except during clinical attacks (Figures 1a and b), confirming our previous study showing a decreased frequency of LCL-specific T cells measured by enzyme-linked immunospot (ELISPOT) assays.^19^ The frequencies of LCL-specific CD3^+^ T cells, CD4^+^ T cells, CD8^+^ T cells, CD4^+^ EM T cells and CD8^+^ EM/EMRA T cells in the PBMC of patients not having a clinical attack (that is, remission, secondary progressive and primary progressive MS) were significantly lower than in healthy EBV-seropositive subjects (Figure 1b). There was a positive correlation between the frequency of LCL-specific CD8^+^ EM/EMRA T cells in the PBMC with the frequency of total CD8^+^ EM/EMRA T cells in the PBMC in patients with MS (*r*=0.30, *P*=0.0004) but not in healthy subjects (*r*=0.04, *P*=0.758; Figure 1c), indicating that the decreased CD8^+^ T-cell response to EBV is associated with the general deficiency of CD8^+^ EM/EMRA T cells that occurs in MS.^13^ In contrast there was no correlation between the frequency of LCL-specific CD4^+^ EM T cells in the PBMC and the frequency of total CD4^+^ EM T cells in the PBMC (data not shown) which is normal in MS.^13^ The increased T-cell response to EBV during attacks of MS was most clearly shown by analysing the frequencies of LCL-specific cells within the different T-cell phenotypes. As shown in Figure 1d, the frequencies of LCL-specific cells within the CD3^+^ T cell, CD4^+^ T cell, CD8^+^ T cell, CD4^+^ EM T cell, CD8^+^ EM T cell, CD8^+^ EMRA T cell and CD8^+^ CM T-cell populations during attacks were all significantly higher than in patients not having an attack (Figure 1d). In contrast to the increased T-cell reactivity to EBV during clinical attacks, the T-cell response to CMV did not increase significantly during clinical attacks (data not shown).

To determine whether the frequency of EBV-specific CD8^+^ T cells is influenced by the expression of HLA-A*02 which protects against MS,^31^ we compared the frequencies of LCL-specific CD8^+^ T cells in the PBMC in HLA-A*02^+^ and HLA-A*02^−^ subjects. There were no significant differences in the median frequencies between HLA-A*02^+^ and HLA-A*02^−^ subjects, either in the healthy control group (0.184% and 0.172%, respectively) or the patients with MS (0.136 and 0.141% respectively), confirming the results of our earlier study using ELISPOT assays to measure the LCL-specific T-cell frequency.^19^ Interestingly, the frequency of LCL-specific T cells within the CD8^+^ population strongly correlated with the frequency of LCL-specific T cells within the CD4^+^ population in MS patients (*r*=0.57, *P*<0.0001), whereas this correlation was weaker in healthy subjects (*r*=0.40, *P*=0.008) (Figure 1e). On multiple linear regression analysis, the slope of the regression line in the MS patients was significantly greater than that in healthy subjects (*P=*0.006).

### The T-cell response to EBV-infected B cells progressively decreases with increasing duration of MS

**Figure 2:**
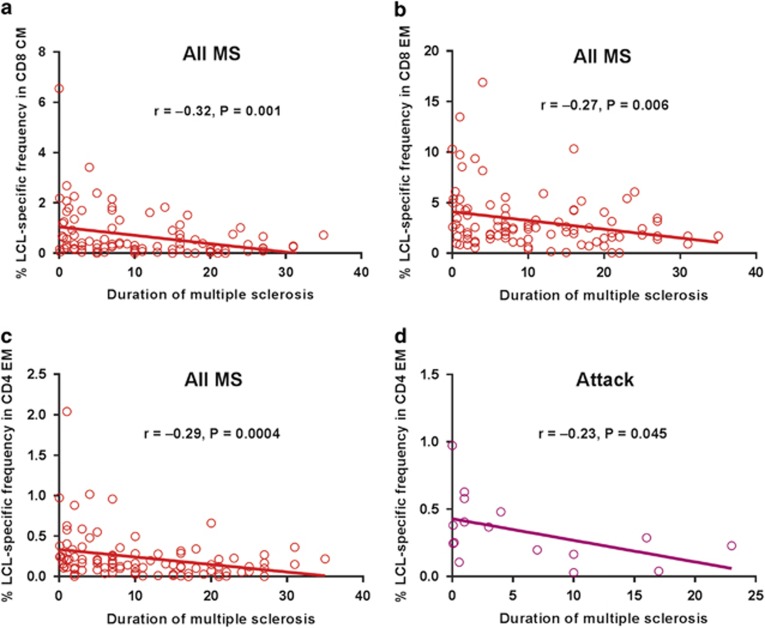
The relationship between the frequency of LCL-specific T cells and duration of MS. (**a**–**c**) In the total group of patients, the percentage of LCL-specific T cells within the CD8^+^ CM population progressively decreased with increasing duration of MS (**a**), as did the frequency of LCL-specific T cells within the CD8^+^ EM population (**b**), and within the CD4^+^ EM population (**c**). (**d**) In patients having an attack the percentage of LCL-specific T cells within the CD4^+^ EM population also declined with increasing disease duration.

The frequency of LCL-specific T cells within the CD8^+^ CM population progressively decreased with increasing duration of MS (*r*=−0.32, *P*=0.001) (Figure 2a), as did the frequency of LCL-specific T cells within the CD8^+^ EM population (*r*=−0.27, *P*=0.006) (Figure 2b) and within the CD8^+^ EMRA population (*r*=−0.16, *P*=0.135). Moreover, the frequency of LCL-specific T cells within the CD4^+^ EM population also declined with increasing disease duration (*r*=−0.29, *P*=0.0004; Figure 2c). These decreases are unlikely to be due to an effect of ageing on the immune system because in healthy EBV-seropositive subjects there was no significant effect of age on the LCL-specific T-cell frequencies (data not shown). Interestingly, even though the frequency of LCL-specific T cells within the CD4^+^ EM population increased substantially during attacks (Figure 1d) it still declined with increasing duration of MS when the analysis was confined to the results during attacks (*r*=−0.23, *P*=0.045), which occurred less frequently over time (Figure 2d), as previously reported.^32^ These results are consistent with progressive T-cell exhaustion of EBV-specific CD4^+^ T cells and CD8^+^ T cells during the course of MS although they could also be due to other factors, for example an age-related decline in the tendency of EBV to reactivate.

### The CD8^+^ T-cell response to EBV lytic phase antigens is reduced at the onset of MS and throughout its course

**Figure 3:**
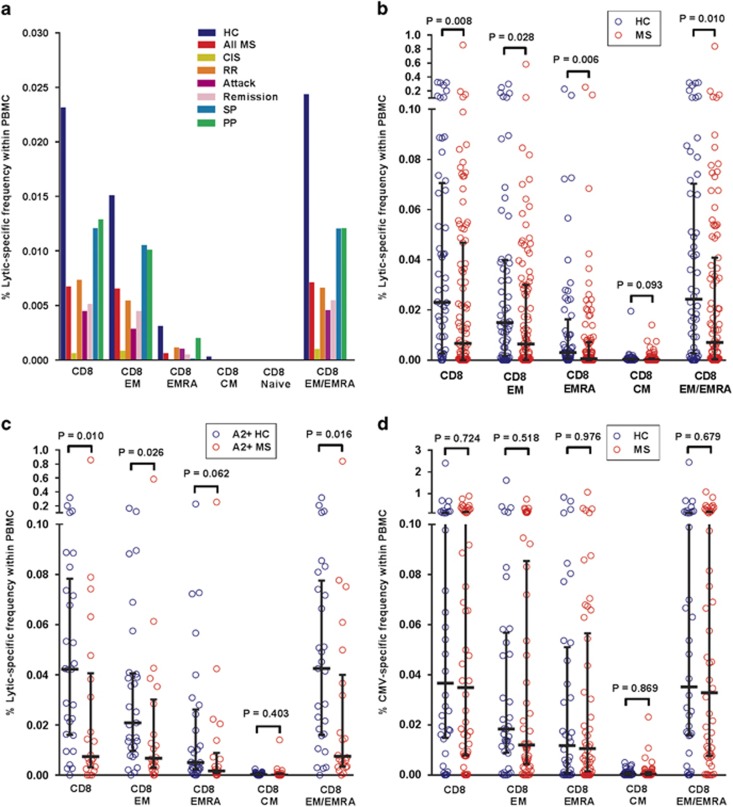
Decreased CD8^+^ T-cell response to EBV lytic phase antigens throughout the course of MS. (**a**) Median percentages of CD8^+^ T cells of different phenotypes producing IFN-γ in response to a pool of HLA-class-I-restricted EBV lytic peptides in the PBMC in EBV-seropositive healthy controls (HC), the total group of MS patients (All MS), patients with clinically isolated syndrome (CIS), patients with relapsing–remitting (RR), secondary progressive (SP) and primary progressive (PP) MS, as well as patients during a clinical attack (Attack) and during remission (Remission). As expected, there was no CD4^+^ T-cell response to these HLA-class-I-restricted peptides. (**b**) Percentages of EBV-lytic-specific CD8^+^ T cells in the PBMC in the total group of patients (MS) compared with healthy controls (HC) (medians with interquartile ranges indicated by black horizontal lines), with bracketed *P* values determined by the Mann–Whitney test. (**c**) Percentages of EBV-lytic-specific CD8^+^ T cells in the PBMC in HLA-A*02^+^ patients (A2+ MS) compared with HLA-A*02^+^ healthy controls (A2+ HC) (medians with interquartile ranges indicated by black horizontal lines), with bracketed *P* values determined by the Mann–Whitney test. (**d**) Percentages of CD8^+^ T cells of different phenotypes producing IFN-γ in response to a pool of HLA-class-I-restricted CMV peptides in the PBMC in CMV-seropositive HC, and CMV-seropositive MS patients (MS) (medians with interquartile ranges indicated by black horizontal lines), with bracketed *P*-values determined by the Mann–Whitney test. There was no CD4^+^ T-cell response.

To study the CD8^+^ T-cell response to EBV lytic phase antigens we used flow cytometry and intracellular cytokine staining to measure the frequencies of CD8^+^ T cells producing IFN-γ in response to a pool of five lytic peptides restricted by common HLA-class I molecules (Table 2). In healthy EBV-seropositive subjects lytic-specific CD8^+^ T cells were predominantly of the EM type and less frequently of the EMRA type (Figure 3a). At all stages of MS, including during clinical attacks, lytic-specific CD8^+^ T cells were decreased in the PBMC (Figure 3a), with the frequencies of lytic-specific CD8^+^ T cells, CD8^+^ EM T cells, CD8^+^ EMRA T cells and CD8^+^ EM/EMRA T cells all being significantly lower than in healthy subjects (Figure 3b). The median frequency of lytic-specific T cells within the CD8^+^ T-cell phenotype in patients with MS (0.047%) was also significantly lower than in healthy subjects (0.118%) (*P=*0.037) (data not shown). Because two of the five peptides in the lytic peptide pool were restricted by HLA-A*02 (Table 2) we wished to ensure that the decreased CD8^+^ T-cell response to the lytic peptide pool was not due to the lower frequency of this allele in patients with MS.^19, 31^ We therefore measured the response to the lytic peptide pool in HLA-A*02^+^ subjects. Figure 3c shows that the frequencies of lytic-specific CD8^+^ T cells, CD8^+^ EM T cells, CD8^+^ EMRA T cells and CD8^+^ EM/EMRA T cells in the PBMC were lower in HLA-A*02^+^ patients than in HLA-A*02^+^ healthy subjects, thus confirming the decreased CD8^+^ T-cell response to EBV lytic phase antigens in MS.

To determine whether the decreased CD8^+^ T-cell response to lytic antigens is limited to EBV infection, we also measured the T-cell response to a pool of 18 HLA-class-I-restricted peptide epitopes of CMV, as a control herpesvirus, in the subjects who were CMV-seropositive. The specificity of the T-cell response to CMV using this approach was demonstrated by the fact that the median frequency of CMV-reactive CD8^+^ T cells in the PBMC was 0.000% in CMV-seronegative healthy subjects, compared with 0.037% in CMV-seropositive healthy subjects (*P*<0.0001). Interestingly, the frequencies of CMV-specific CD8^+^ T cells in the 50 CMV-seropositive MS patients did not differ significantly from those of the 36 CMV-seropositive healthy subjects, either in the PBMC (*P*=0.724) (Figure 3d) or within the total CD8^+^ population, whereas in these CMV-seropositive subgroups the frequency of EBV-lytic-specific CD8^+^ T cells in the PBMC was still significantly lower in the MS patients than in the healthy subjects (*P*=0.021). These results indicate impaired CD8^+^ T-cell control of EBV, but not CMV, reactivation in MS.

### Skewing of the EBV-specific CD8^+^ T-cell response in MS away from lytic to latent antigens

**Figure 4:**
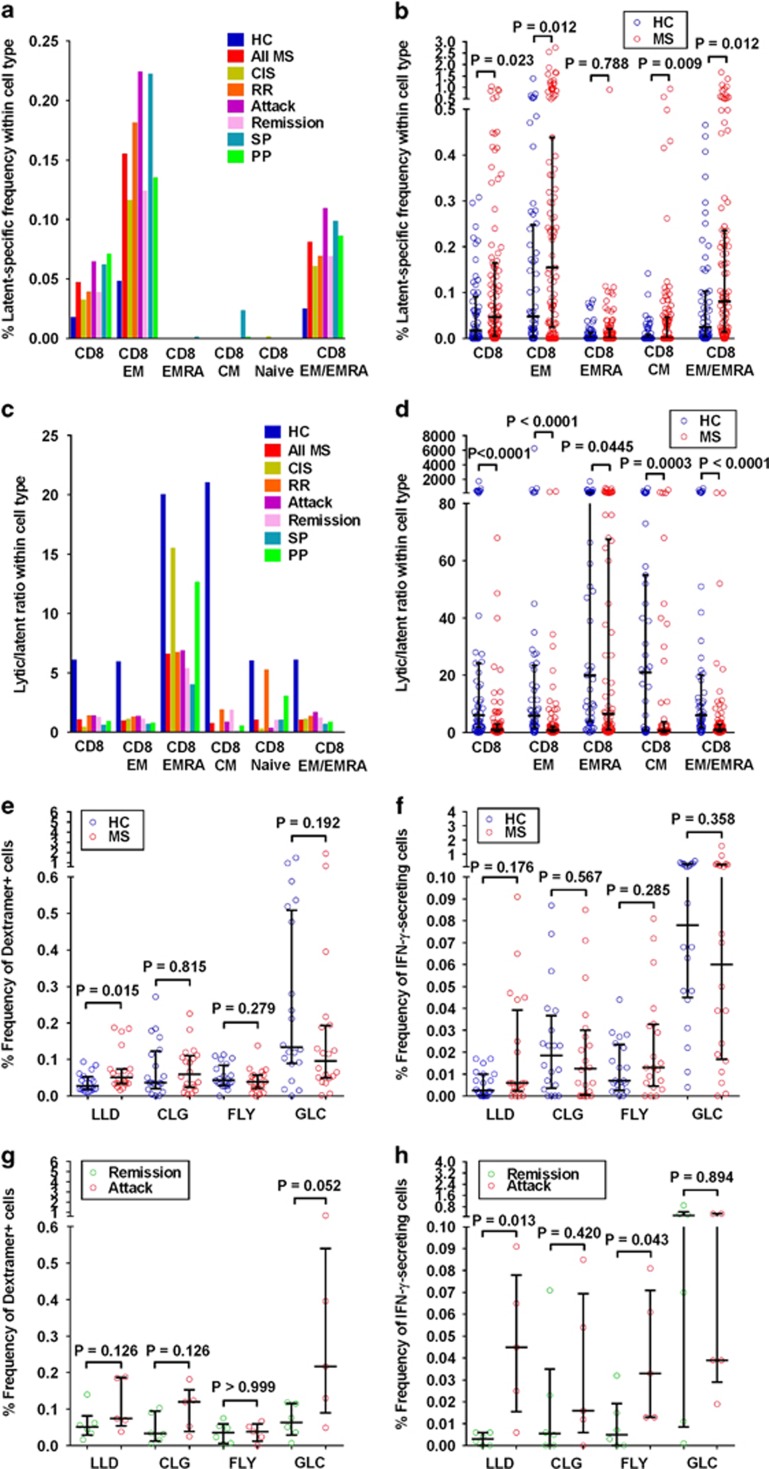
Skewing of the EBV-specific CD8^+^ T-cell response in MS away from lytic to latent antigens. (**a**) Median percentages of CD8^+^ T cells within the different phenotypes producing IFN-γ in response to a pool of HLA-class-I-restricted EBV latent peptides in EBV-seropositive healthy controls (HC), the total group of MS patients (All MS), patients with clinically isolated syndrome (CIS), patients with relapsing–remitting (RR), secondary progressive (SP) and primary progressive (PP) MS, as well as patients during a clinical attack (Attack) and during remission (Remission). As expected, there was no CD4^+^ T-cell response to these HLA-class-I-restricted peptides. (**b**) Percentages of latent-specific cells within the CD8^+^, CD8^+^ EM, CD8^+^ EMRA, CD8^+^ CM and CD8^+^ EM/EMRA T-cell populations in patients with MS (MS) compared with healthy controls (HC) (medians with interquartile ranges indicated by black horizontal lines), with bracketed *P*-values determined by the Mann–Whitney test. (**c**) Median lytic/latent ratio within the different phenotypes in EBV-seropositive HC, the total group of MS patients (All MS), patients with CIS, patients with RR, SP and PP MS, as well as patients during a clinical attack (Attack) and during remission (Remission). For each subject the ratio was calculated by dividing the frequency of CD8^+^ T cells producing IFN-γ in response to pooled lytic peptides by the frequency of CD8^+^ T cells producing IFN-γ in response to pooled latent peptides. (**d**) Lytic/latent ratio within the CD8^+^, CD8^+^ EM, CD8^+^ EMRA, CD8^+^ CM and CD8^+^ EM/EMRA T-cell populations in the total group of MS patients (MS) compared with HC (medians with interquartile ranges indicated by black horizontal lines), with bracketed *P* values determined by the Mann–Whitney test. (**e** and **f**) Percentages of T cells specific for individual EBV latent (LLD, CLG and FLY) and lytic (GLC) peptides in the CD8^+^ population in 20 HLA-A*02^+^ EBV-seropositive healthy subjects (HC) and 20 HLA-A*02^+^ MS patients (MS) measured by binding to peptide-HLA-A*02 Dextramers (**e**) and IFN-γ production (**f**) (medians with interquartile ranges indicated by black horizontal lines), with bracketed *P-*values determined by the Mann–Whitney test. (**g** and **h**) Percentages of T cells specific for individual EBV latent and lytic peptides in the CD8^+^ population in HLA-A*02^+^ patients during clinical attacks (Attack) or during remission (Remission) measured by binding to peptide-HLA-A*02 Dextramers (**g**) and IFN-γ production (**h**) (medians with interquartile ranges indicated by black horizontal lines), with bracketed *P*-values determined by the Mann–Whitney test.

To study the CD8^+^ T-cell response to EBV latent phase antigens we measured the frequencies of CD8^+^ T cells producing IFN-γ in response to a pool of 13 latent peptides restricted by common HLA-class I molecules (Table 2). In healthy EBV-seropositive subjects latent-specific CD8^+^ T cells were almost entirely of the EM type with very few cells of the EMRA type (Figure 4a). In contrast to the reduced EBV-lytic-specific CD8^+^ T-cell response, the frequency of latent-specific CD8^+^ T cells was increased in MS. The increased CD8^+^ T-cell response to EBV latent antigens was most clearly shown by analysing the frequencies of latent-specific cells within the different T-cell phenotypes (Figure 4a). As shown in Figure 4b, the frequencies of latent-specific cells within the CD8^+^ T cell, CD8^+^ EM T cell, CD8^+^ CM T cell and CD8^+^ EM/EMRA T-cell populations were all significantly higher in patients with MS than in healthy EBV-seropositive subjects.

To determine the relative contributions of the latent and lytic CD8^+^ T-cell responses to the total EBV-specific CD8^+^ T-cell response, we calculated the lytic/latent ratio in each subject by dividing the frequency of CD8^+^ T cells producing IFN-γ in response to pooled lytic peptides by the frequency of CD8^+^ T cells producing IFN-γ in response to pooled latent peptides. This approach minimizes the effect of the heterogeneity of the HLA-class I genes among the different subjects. In healthy EBV-seropositive subjects the response to lytic peptides was much greater than the response to latent peptides (Figure 4c). At all stages of MS, including during clinical attacks, the CD8^+^ T-cell response was markedly shifted away from lytic antigens to latent antigens (Figure 4c). The median lytic/latent ratio in the CD8^+^ T-cell population was only 1.03 in patients with MS compared with 6.07 in healthy subjects (*P*<0.0001) (Figure 4d). The median lytic/latent ratios in the CD8^+^ EM T cell, CD8^+^ EMRA T cell, CD8^+^ CM T cell and CD8^+^ EM/EMRA T-cell subpopulations were also significantly lower in MS than in healthy subjects (Figure 4d). Furthermore, when we confined the analysis to HLA-A*02^+^ subjects the median lytic/latent ratio in the CD8^+^ T-cell population was only 1.38 in patients with MS compared with 9.31 in HLA-A*02^+^ healthy subjects (*P*=0.002) (data not shown). Thus in MS there is a marked skewing of the EBV-specific CD8^+^ T-cell response away from the normal predominant lytic antigen response to a major latent antigen response.

We also determined the frequencies of CD8^+^ T cells specific for individual EBV peptides in 20 HLA-A*02^+^ healthy subjects and 20 HLA-A*02^+^ MS patients by using flow cytometry to detect T cells binding to selected HLA-peptide complexes stabilized on a dextran polymer backbone with attached fluorophores (Dextramers) and, in a different tube of cells from the same sample, intracellular cytokine staining to measure the frequencies of T cells producing cytokines in response to stimulation with the corresponding peptides. Consistent with the increased frequency of CD8^+^ T cells responding to pooled latent peptides in MS, the median frequency of cells binding the LLD-Dextramer was significantly higher in patients than in EBV-seropositive healthy subjects (Figure 4e). We also calculated the lytic/latent ratio in each subject by dividing the frequency of T cells binding the lytic GLC-Dextramer by the frequency of T cells binding the latent LLD-Dextramer. The median GLC/LLD ratio in patients with MS (1.75) was significantly lower than in healthy subjects (6.38) (*P*=0.014) (data not shown), thus confirming our findings using pooled peptides. Figure 4f shows that the median frequency of T cells secreting IFN-γ in response to stimulation with the EBNA3C-derived LLD peptide was higher in patients than in controls. During attacks the frequencies of T cells binding two of the latent Dextramers increased, although not significantly (Figure 4g), and the frequencies of cells secreting IFN-γ in response to stimulation with each of the three individual latent peptides increased, two significantly so (Figure 4h). During attacks the proportion of EM cells increased markedly in the LLD-Dextramer^+^ population (41% compared with 7% in the patients in remission), as it did to a lesser extent in the CLG-Dextramer^+^ population (54% compared with 26% in the patients in remission) (data not shown). Although the number of T cells binding the lytic GLC-Dextramer also increased during attacks (Figure 4g), there was no corresponding increase in the number of T cells producing IFN-γ in response to the GLC peptide (Figure 4h), suggesting impaired function of these cells.

### CD8^+^ T cells recognizing EBV latent phase antigens in MS show T-cell exhaustion

**Figure 5:**
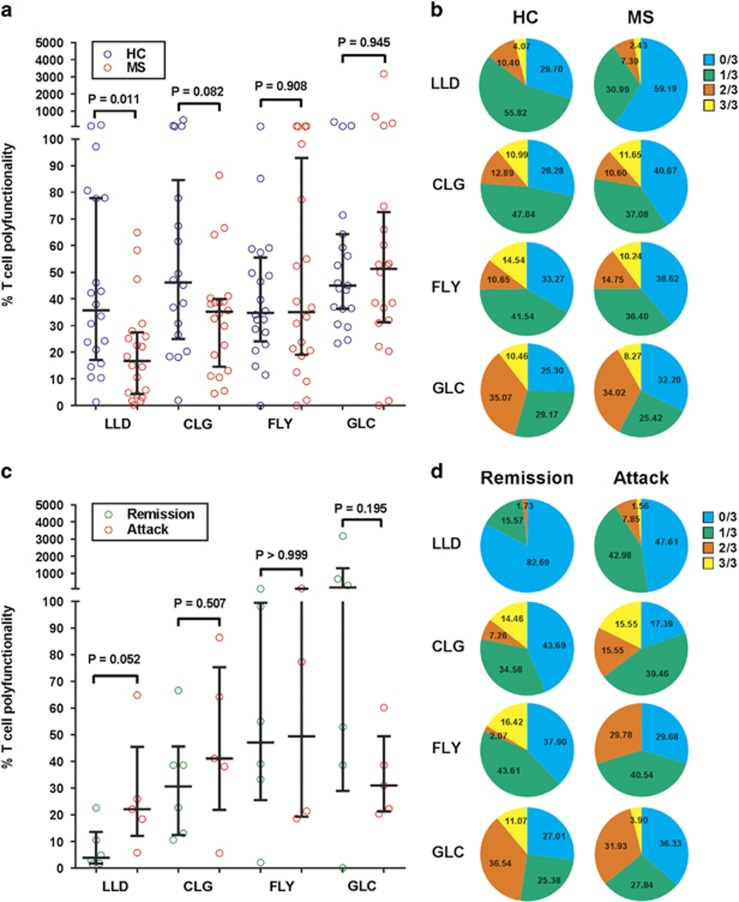
EBV-specific CD8^+^ T-cell polyfunctionality in MS. (**a** and **b**) Polyfunctionality of CD8^+^ T cells specific for individual EBV latent (LLD, CLG and FLY) and lytic (GLC) peptides in 20 HLA-A*02^+^ EBV-seropositive healthy subjects (HC) and 20 HLA-A*02^+^ MS patients (MS) determined by measuring the frequencies of T cells producing IL-2, TNF-α and IFN-γ in response to stimulation with each peptide and, in a different tube of cells, the frequencies of T cells binding to the respective peptide-HLA-A*02 Dextramer. (**a**) Ratio of the polyfunctionality index to the percentage of T cells binding the respective peptide-HLA-A*02 Dextramer in the CD8^+^ population (medians with interquartile ranges indicated by black horizontal lines), with bracketed *P*-values determined by the Mann–Whitney test. (**b**) Pie charts showing the mean percentages of Dextramer^+^ CD8^+^ T cells producing zero, one, two or three cytokines. (**c** and **d**) Polyfunctionality of CD8^+^ T cells specific for individual EBV latent and lytic peptides in HLA-A*02^+^ MS patients during clinical attacks (Attack) or during remission (Remission). (**c**) Ratio of the polyfunctionality index to the percentage of T cells binding the respective peptide-HLA-A*02 Dextramer in the CD8^+^ population (medians with interquartile ranges indicated by black horizontal lines), with bracketed *P-*values determined by the Mann–Whitney test. (**d**) Pie charts showing the mean percentages of Dextramer^+^ CD8^+^ T cells producing zero, one, two or three cytokines.

CD8^+^ T-cell exhaustion occurs in high-grade chronic viral infections and is manifested as a loss of function occurring in a hierarchical manner, with the exhausted cells initially failing to produce IL-2, later failing to produce TNF-α and then IFN-γ and eventually dying.^33, 34^ To assess T-cell exhaustion we used flow cytometry and intracellular cytokine staining to measure the frequencies of T cells producing IL-2, TNF-α and IFN-γ in response to stimulation with selected HLA-A*02-restricted peptides and, in a different tube of cells from the same sample, the frequencies of T cells binding to the respective peptide/HLA-A*02 Dextramers in 20 HLA-A*02^+^ healthy subjects and 20 HLA-A*02^+^ MS patients. To assess T-cell polyfunctionality we calculated the polyfunctionality index,^35^ which gives successively higher weightings to the frequencies of cells producing one, two and three cytokines; we then divided this index by the frequency of T cells binding the respective HLA-peptide Dextramer. Although T cells binding the latent LLD-Dextramer or latent CLG-Dextramer were more frequent in patients with MS than in healthy EBV carriers (Figure 4e) the polyfunctionality of these cells, especially that of the LLD-specific cells, was impaired (Figure 5a), indicating T-cell exhaustion, although there was no associated increase in PD-1 expression (data not shown). Furthermore, the proportions of LLD-specific and CLG-specific cells producing no cytokines were higher in patients than controls (Figure 5b). Interestingly, during attacks the polyfunctionality of the latent-specific, especially the LLD-specific, T cells increased but that of the lytic-specific cells decreased (Figure 5c). As shown in the pie charts in Figure 5d, the proportions of latent-specific T cells producing no cytokines decreased during attacks whereas that of the lytic-specific T cells increased.

### Relationships of EBV genome load and anti-EBV antibody titres with the frequency of EBV-specific CD8^+^ T cells in MS

**Figure 6:**
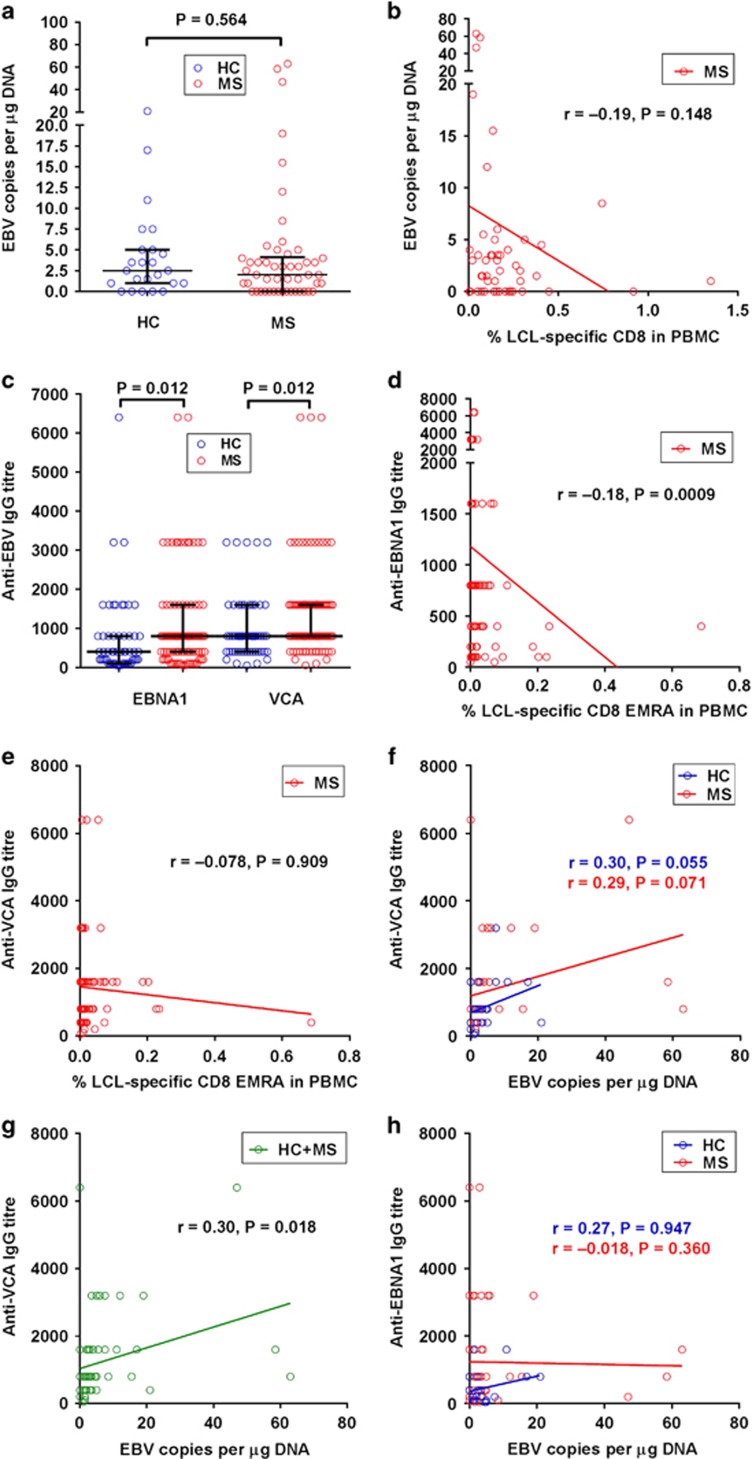
The relationships of EBV genome load and anti-EBV antibody titres with the frequency of EBV-specific CD8^+^ T cells in MS. (**a**) EBV DNA copy number in the PBMC in healthy EBV-seropositive subjects (healthy controls (HC)) and patients with MS (MS) (medians with interquartile ranges indicated by black horizontal lines), with *P-*value determined by the Mann–Whitney test. (**b**) Relationship between the EBV genome load and the LCL-specific CD8^+^ T-cell frequency in MS patients. (**c**) Titres of anti-EBNA1 IgG and anti-VCA IgG in the sera of healthy EBV-seropositive subjects (HC) and patients with MS (MS) (medians with interquartile ranges indicated by black horizontal lines), with bracketed *P-*values determined by the Mann–Whitney test. (**d**) Relationship between the anti-EBNA1 IgG titre and the LCL-specific CD8^+^ EMRA T-cell frequency in the PBMC in MS patients. (**e**) Relationship between the anti-VCA IgG titre and the LCL-specific CD8^+^ EMRA T-cell frequency in the PBMC in MS patients (**f**) Relationship between the anti-VCA IgG titre and the EBV genome load in HC and patients with MS (MS). (**g**) Relationship between the anti-VCA IgG titre and the EBV genome load in the combined groups of HC and patients with MS. (**h**) Relationship between the anti-EBNA1 IgG titre and the EBV genome load in HC and patients with MS.

To investigate the relationship between the EBV DNA load in the blood and the EBV-specific T-cell response, we employed real-time PCR with primers and probe directed towards a conserved portion of the *BamH1W* segment of the EBV genome^36^ in DNA extracted from the same PBMC sample used to study the T-cell response in 24 of the healthy EBV-seropositive subjects and 50 of the MS patients. The median EBV DNA copy number in the PBMC in the patients (2 μg^−1^ of DNA) did not differ significantly from that in the healthy controls (2.5 μg^−1^ of DNA) (*P*=0.564) (Figure 6a), consistent with most previous studies.^2^ In the patients with MS the EBV load increased as the LCL-specific CD8^+^ T-cell frequency decreased (Figure 6b), although the correlation did not reach statistical significance (*r*=−0.19, *P*=0.148).

To investigate the relationship between anti-EBV antibody levels and the EBV-specific CD8^+^ T-cell response and EBV load we used ELISA to measure anti-EBV antibodies in the serum in 55 out of the 56 EBV-seropositive healthy subjects and 94 out of the 95 MS patients in whom the T-cell response was studied. We detected IgG specific for EBV viral capsid antigen (VCA) in 98% of the healthy controls and 99% of the patients, and anti-EBNA1 IgG in 93% of the controls and 96% of the patients; all subjects were seropositive for one or both antibodies. Anti-VCA IgA was present in 0% of controls and 6% of patients (*P*=0.085). Anti-VCA IgM, IgG specific for EBV early antigen (EA), anti-EA IgM and anti-EA IgA were each detected in 0–6% of subjects, with no significant differences between controls and patients. As expected, the titres of anti-EBNA1 IgG and anti-VCA IgG were increased in MS (Figure 6c). Interestingly, the anti-EBNA1 IgG titre correlated inversely with the frequency of LCL-specific CD8^+^ T cells, particularly CD8^+^ EMRA T cells, in the PBMC in the patients with MS (*r*=−0.18, *P*=0.0009) (Figure 6d). The anti-EBNA1 IgG titre also correlated inversely with the frequency of EBV-lytic-specific CD8^+^ EMRA T cells in the PBMC of patients (*r*=−0.11, *P*=0.015) (data not shown). In contrast, the anti-VCA IgG titre did not correlate with the EBV-specific CD8^+^ EMRA T-cell response (Figure 6e). However, the anti-VCA IgG titre did correlate positively with the EBV DNA load in the blood in both healthy subjects (*r*=0.30, *P*=0.055) and patients with MS (*r*=0.29, *P*=0.071; Figure 6f), with the correlation reaching statistical significance when the results of both groups of subjects were combined (*r*=0.30, *P*=0.018; Figure 6g). On the other hand there was no correlation between the anti-EBNA1 IgG titre and the EBV DNA load in the blood (Figure 6h).

## Discussion

**Figure 7:**
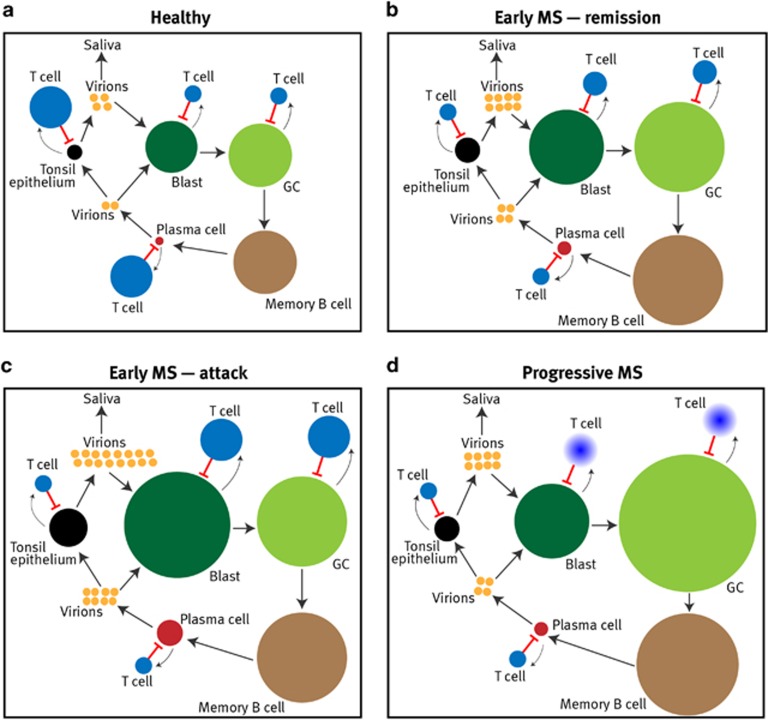
Proposed model of defective CD8^+^ T-cell control of EBV infection in MS. In healthy EBV carriers, (**a**) there is a dynamic equilibrium between the EBV-infected cell populations and the T-cell response. EBV-specific CD8^+^ T cells (T cell) exert a key role in controlling EBV infection by killing infected cells in the B blast, germinal centre (GC) B cell, plasma cell and tonsil epithelial cell, but not memory B cell, populations. The large arrows indicate the cycle of EBV infection: virion→B blast→GC B cell→memory B cell→plasma cell→virion→epithelial cell→virion→B blast. Smaller arrows indicate stimulation of T cells by EBV antigens from the infected populations. The relative sizes of the different EBV-infected cell populations are indicated by the circle sizes, based on the study by Hawkins *et al.*^6^ The relative sizes of the EBV-specific CD8^+^ T-cell populations are also indicated by the circle sizes; however, it is important to note that the EBV-specific CD8^+^ T-cell population is several orders of magnitude larger than the EBV-infected cell population, a distinction not depicted here. For simplicity, the EBV-specific CD4^+^ T-cell population and anti-EBV antibody response are not shown. At all stages of MS (**b**–**d**) the EBV-lytic-specific CD8^+^ T-cell population is decreased, allowing increased production of virions which infect naive B cells driving them into the blast phase. The resultant expansion of the infected blast population stimulates EBV-latent-specific CD8^+^ T cells which proliferate and restrict this expansion, but not without increased flow out of infected blast cells into a consequently enlarged EBV-infected GC cell population, which in turn is partially controlled by the augmented EBV-latent-specific CD8^+^ T-cell population. In the same way the EBV-infected memory B cell pool also grows, as does the population of plasma cells reactivating EBV infection. During clinical attacks of MS (**c**) there is increased differentiation of EBV-infected memory B cells into lytically infected plasma cells as a result of the various microbial infections that trigger attacks of MS. This EBV reactivation is inadequately regulated by the already deficient EBV-lytic-specific CD8^+^ T-cell response, resulting in increased virion production and increased infection of the blast pool, this in turn stimulating proliferation of the EBV-latent-specific CD8^+^ T-cell population which restricts further growth of the infected blast population. In progressive MS (**d**) the EBV-latent-specific CD8^+^ T-cell response becomes exhausted (indicated by fading), resulting in unchecked expansion of the infected GC population and the development of EBV-infected lymphoid tissue in the CNS.

In this comprehensive study of the T-cell response to EBV in MS, we have shown that there is a decreased CD8^+^ T-cell response to EBV lytic phase antigens at the onset of MS and at all subsequent stages of the disease. In contrast to the decreased response to the lytic antigens of EBV, the CD8^+^ T-cell response to the lytic antigens of CMV, another common human herpesvirus, was normal in patients with MS. Even in healthy EBV carriers, EBV is continuously being reactivated in the tonsil with shedding of virions into saliva,^5^ a process normally kept in check by lytic-specific cytotoxic CD8^+^ T cells (Figure 7a).^7, 8^ Thus the predicted consequence of defective CD8^+^ T-cell control of EBV reactivation is increased production of infectious virions with increased oral shedding, as occurs in children with MS,^37^ and increased infection of new naive B cells, which become blasts in a new cycle of infection (Figures 7b and d).^6^ Indeed the amplification of virus production in the tonsil wherein virus released from each bursting plasma cell infects epithelial cells which in turn generate enough virions to infect ~10 000 naive B cells^6^ means that impaired immune control of this critical stage of the EBV life cycle can have a major follow-on effect into the infected blast population. The crucial importance of controlling the lytic phase of infection is also supported by the study of Hopwood *et al.*^38^ who concluded that in healthy individuals viral loads are maintained within normal limits by cytotoxic CD8^+^ T cells directed against lytic rather than latent EBV proteins. According to the mathematical model of EBV infection proposed by Hawkins *et al.*,^6^ increased input into the EBV-infected blast pool from the lytic phase will increase the size of the blast population, in turn stimulating the expansion of CD8^+^ T cells specific for EBV latent antigens, as we found in patients with MS. If the increase in latent-specific cytotoxic CD8^+^ T cells suffices to counteract the increased input into the blast population, the size of the infected blast cell population remains at its pre-existing healthy size at the expense of a sustained increase in the latent-specific CD8^+^ T-cell population. However, if the persistently high EBV latent antigen load induces T-cell exhaustion of the latent-specific T cells, as occurs in high-grade chronic viral infections,^33, 34^ the increased input into the blast pool from the lytic phase is no longer counterbalanced by the T-cell response, so that the blast population increases in size, as does its input into the germinal centre pool. We have shown that there is indeed T-cell exhaustion of CD8^+^ T cells recognizing EBV latent antigens in MS. Interestingly the T-cell exhaustion appears preferentially to affect CD8^+^ T cells responding to the EBNA3C-derived LLD peptide rather than the LMP2A-derived CLG and FLY peptides. Potential explanations for the predilection of EBNA3C-specific CD8^+^ T cells to exhaustion include their immunodominance^8^ and the earlier expression of EBNA3 proteins than LMP proteins after infection of naive B cells.^39^ Increased input of cells from the expanded EBV-infected blast pool into the germinal centre pool will enlarge the infected germinal centre B-cell population, which will continue to grow if inadequately controlled. Furthermore, unchecked growth of the EBV-infected germinal centre B-cell pool will increase the infected memory B-cell population and thus the number of latently infected cells available to reactivate the virus into the lytic stage,^6^ thereby further taxing the already impaired EBV-lytic-specific CD8^+^ T-cell response. This in turn leads to increased generation of virions which infect naive B cells, driving them into clonal expansion with further enlargement of the EBV-infected blast population—truly a vicious cycle. Progressive exhaustion of EBV-latent-specific T cells will permit unbridled proliferation of EBV-infected germinal centre B cells (Figure 7d), which might underlie the development of EBV-infected lymphoid follicles in the brain in secondary progressive MS.^40^

Our finding that the EBV DNA copy number in the blood is not increased in patients with MS is consistent with most previous studies.^2^ The limitation of this measurement as an index of the total body EBV load in MS stems from the following two factors. First, because it does not discriminate between DNA in virions and viral DNA in latently infected cells it does not actually measure the frequency of EBV-infected B cells in the blood, which is probably increased in patients with MS, as suggested by increased spontaneous EBV-induced transformation of peripheral blood B cells.^41, 42, 43^ Second, the EBV DNA copy number in the blood does not reflect the EBV load in compartments where it is increased in MS, namely the brain, as indicated by elevated titres of anti-EBV antibodies in the cerebrospinal fluid^24^ and the detection of EBV in brain tissue,^40, 44, 45^ and in the oral mucosa.^37^

During clinical attacks of MS there was expansion of both the EBV-specific CD4^+^ T-cell and EBV-specific CD8^+^ T-cell populations, with increased functionality of latent-specific, and decreased functionality of lytic-specific, CD8^+^ T cells. The increased T-cell response to EBV-infected B cells during attacks is consistent with the study of Latham *et al.*^46^ who reported an increased LCL-specific T-cell response preceding increased disease activity, as determined by magnetic resonance imaging. Our finding of an increase in the frequency of CD8^+^ T cells binding the GLC-Dextramer derived from the lytic protein BMLF1 during attacks confirms the results of Angelini *et al.*^27^ who found an increased frequency of CD8^+^ T cells binding HLA-class I pentamers of EBV lytic peptides during the active phase of MS; however, by also examining cytokine production by these lytic-specific T cells we were able to show that their function actually decreased during attacks whereas latent-specific T cells increased in both number and polyfunctionality. The increased proportion of EM cells within the LLD-Dextramer^+^ and CLG-Dextramer^+^ CD8^+^ T-cell populations during attacks indicates expansion of activated effector cells specific for EBV latent antigens. Because infections caused by a wide variety of microbial agents can both trigger attacks of MS^47, 48, 49^ and reactivate EBV infection,^50, 51, 52, 53, 54^ we hypothesize that expansion of EBV-specific CD4^+^ T cells and CD8^+^ T cells during clinical attacks occurs in response to reactivation of EBV in peripheral lymphoid organs by the intercurrent infections triggering the attacks. We propose that this EBV reactivation is poorly controlled by EBV-lytic-specific CD8^+^ T cells, leading to increased production of virus which infects additional naive B cells, thereby expanding the pool of EBV-infected B blasts and increasing the proportion of T cells within the total T-cell population responding to EBV latent antigens (Figure 7c). Early in the course of MS an abrupt increase in the EBV-infected blast population during intercurrent infections might be able to stimulate proliferation of EBV-latent-specific CD4^+^ T cells and CD8^+^ T cells, but with increasing duration of MS the capacity of these T cells to respond diminishes, as shown for the EBV-specific CD4^+^ T-cell population in Figure 2d, the diminution occurring as a result of T-cell exhaustion and possibly other factors, for example an age-related decline in the tendency of EBV to reactivate. Because myelin-reactive T cells also increase in the blood during MS attacks,^55, 56^ it is vital to determine whether CNS-specific T cells, EBV-specific T cells or both enter the CNS during attacks and initiate inflammation, demyelination and axonal transection.

With increasing duration of MS we observed a progressive decrease in the frequency of EBV-specific T cells in the CD8^+^ population, confirming the finding of Jilek *et al.*^26^ The decrease particularly involved EBV-specific CD8^+^ CM T cells and CD8^+^ EM T cells and was accompanied by a decrease in the frequency of EBV-specific T cells within the CD4^+^ EM T-cell population. CD4^+^ T cell help is necessary for the optimal expansion of CD8^+^ T cells responding to EBV infection.^57^ We found that the correlation between the EBV-specific CD4^+^ T-cell frequency and the EBV-specific CD8^+^ T-cell frequency was stronger in MS than in healthy EBV carriers, in accordance with the finding of West *et al.*^58^ that, in the setting of a sustained high viral load, memory CD8^+^ T cells have an increased reliance on CD4^+^ T cell help. We postulate that the progressive decline in EBV-specific T cells with increasing duration of MS reflects increasing T-cell exhaustion, first affecting CD4^+^ T cells and then involving CD8^+^ T cells, and that this exhaustion further increases the viral load. Future studies should examine the role of EBV-specific CD4^+^ T cells in MS in more detail, for example by measuring HLA-Class II tetramer responses to EBV lytic and latent epitopes. In the present study, we used EBV-infected LCL as a surrogate for EBV-infected cells *in vivo*; however, it is possible that LCL do not represent all aspects of EBV infection *in vivo*, for example infection of epithelial cells in the tonsil and of B cells and plasma cells in the CNS environment.

What is the primary cause of the decreased CD8^+^ T-cell response to EBV lytic phase antigens at the onset of MS and throughout the disease course? The positive correlation between the frequency of LCL-specific CD8^+^ EM/EMRA T cells in the blood and the frequency of total CD8^+^ EM/EMRA T cells in the blood in MS indicates that the decreased CD8^+^ T-cell response to EBV is associated with the general deficiency of CD8^+^ EM/EMRA T cells that occurs early in the disease and persists throughout its course.^13^ In view of the critical role of type I IFN (IFN-α and/or IFN-β) in the generation of CD8^+^ T-cell memory,^59^ one possible mechanism for decreased CD8^+^ T-cell memory in MS is the diminished production of type I IFN that occurs in this disease.^60, 61^ Another important question is why the CD8^+^ T-cell deficiency impairs the response to EBV but not to CMV. EBV differs from CMV and the other herpesviruses in being able to amplify the viral load *in vivo* by inducing clonal expansion of latently infected cells, with EBNA1 maintaining a stable number of EBV genomes within dividing cells.^3^ This proliferation of latently infected cells increases the number of cells available to reactivate the virus into the lytic stage, contributing to the perpetual EBV reactivation in healthy EBV carriers^5^ and increasing the demand on the EBV-lytic-specific CD8^+^ T-cell response. It is also possible that, in response to CMV infection, people with MS are able to overcome the general CD8^+^ EM/EMRA T-cell deficiency by augmenting the proportion of CMV-specific T cells within the decreased total CD8^+^ EM/EMRA T-cell population, in the same way that healthy subjects maintain an inflated memory CD8^+^ T-cell response to CMV.^62^ Although the CD8^+^ T-cell response to CMV appears to be normal, the increased frequency of herpes zoster infection in people who develop MS^63, 64, 65, 66^ raises the possibility of impaired CD8^+^ T-cell control of herpes zoster. Further studies are needed to measure the T-cell response to herpes zoster and the other herpesviruses in MS. It is also not clear why the CD8^+^ T-cell defect in MS initially affects the response to EBV lytic antigens rather than latent antigens. One possible explanation is that, given the massive amplification of lytic infection in tonsil epithelial cells,^5, 6^ healthy regulation of EBV infection requires a faster CD8^+^ T-cell response to lytic antigens than to latent antigens. If this is the case, the delayed presentation of CD4^+^ T-cell epitopes compared with CD8^+^ T-cell epitopes^67^ might limit CD4^+^ T cell help to EBV-lytic-specific CD8^+^ T cells at the beginning of a sudden surge of EBV reactivation.

A hallmark of MS is the elevation of serum IgG antibodies against a wide range of EBV lytic and latent proteins, including VCA and EBNA1.^2^ Indeed, serum anti-EBNA1 IgG is increased, not only at the onset,^68^ but even before the onset of the disease.^69, 70, 71, 72^ As expected, the titres of anti-EBNA1 IgG and anti-VCA IgG were increased in our patients. The inverse correlation between the anti-EBNA1 IgG titre and the frequency of LCL-specific CD8^+^ T cells in MS confirms our previous finding of an inverse correlation between this titre and the frequency of LCL-specific T cells, as determined by ELISPOT assays,^19^ suggesting that the elevated anti-EBNA1 IgG titre reflects an increased EBV latent antigen load resulting from the defective T-cell control of EBV. Notably, the inverse correlation was strongest for that between the anti-EBNA1 IgG titre and the frequency of LCL-specific CD8^+^ EMRA T cells, which, as shown in Figures 3a and 4a, predominantly consist of cells specific for EBV lytic phase antigens. Coupled with the inverse correlation between the anti-EBNA1 IgG titre and the frequency of EBV-lytic-specific CD8^+^ EMRA T cells, this supports our proposal that defective CD8^+^ T-cell control of EBV reactivation leads to increased infection of new naive B cells, generating an expanded population of EBV-infected blasts and increased EBNA1 antigen load, which stimulates production of anti-EBNA1 IgG. The lack of an inverse correlation between the anti-VCA IgG titre and the LCL-specific EMRA CD8^+^ T-cell frequency might be explained as follows: the increased lytic antigen load resulting from defective CD8^+^ T-cell regulation of EBV reactivation would stimulate not only anti-VCA IgG production but also increased, albeit still inadequate, proliferation of lytic-specific CD8^+^ T cells, with this direct relationship tending to negate any inverse relationship between the lytic-specific CD8^+^ T-cell response and anti-VCA IgG production. We found a positive correlation between the anti-VCA, but not the anti-EBNA1, IgG titre and EBV DNA load in the blood in both healthy subjects and patients with MS, as reported for patients with Hodgkin’s lymphoma and their healthy relatives.^73^

In conclusion, we have shown that patients with MS have defective T-cell control of EBV infection which might underlie the accumulation of EBV-infected B cells in the CNS^40^ and subsequent development of the disease. We have proposed a model (Figure 7) where decreased CD8^+^ T-cell control of EBV reactivation permits increased production of virus and consequent expansion of the latently infected B-cell population. To test this model we suggest that further studies are necessary to determine: (i) the cause of CD8^+^ EM/EMRA T-cell deficiency in MS, whether it genetically determined, as we have previously hypothesized^10^ and related to decreased type I IFN production; (ii) whether CD8^+^ T-cell deficiency precedes the onset of MS and is present in healthy first-degree relatives of people with MS, as in the healthy relatives of people with Sjögren’s syndrome;^74^ (iii) whether sunlight deprivation and vitamin D deficiency aggravate the CD8^+^ T-cell deficiency, as previously postulated;^10^ (iv) how and why the EBV-specific CD4^+^ T-cell response declines during the course of MS; (v) whether oral shedding of EBV is increased during clinical attacks; (vi) whether the frequency of EBV-infected memory B cells in the blood is increased in MS, as in rheumatoid arthritis^75^ and systemic lupus erythematosus;^76^ (vii) whether EBV-infected B cells and plasma cells in the CNS in MS are autoreactive, as are EBV-infected plasma cells in the target organs of rheumatoid arthritis^77^ and Sjögren’s syndrome;^78^ and (viii) finally and most importantly, whether therapies aimed at controlling EBV infection, such as EBV-specific T-cell therapy,^79^ prevent and cure MS.

## Methods

### Patients and controls

This study was approved by the Royal Brisbane and Women’s Hospital Human Research Ethics Committee and The University of Queensland Medical Research Ethics Committee. Blood (60 ml) was collected from 95 patients with MS and 56 EBV-seropositive healthy subjects following informed consent. The demographic and clinical details of the healthy subjects and patients with MS are presented in Table 1. The patient group included eight patients with the first attack of the type seen in MS (clinically isolated syndrome), 33 with relapsing–remitting MS, 31 with secondary progressive MS and 23 with primary progressive MS. Out of the 41 patients with clinically isolated syndrome or relapsing–remitting MS, 16 had had a clinical attack within 30 days of venesection, and 25 were in remission. All patients with relapsing–remitting MS, secondary progressive MS and primary progressive MS met the 2005 (ref. 80) and/or the 2010 Revised McDonald Criteria for a diagnosis of MS.^81^ The patients had not received corticosteroids or immunomodulatory therapy for at least 3 months before venesection. Twenty of the MS patients had previously received at least one immunomodulatory drug (other than corticosteroids), most frequently interferon-β or glatiramer acetate, and less frequently azathioprine (two patients), methotrexate (three patients), natalizumab (one patient) and sulphasalazine (for polyarthropathy) (one patient) (Supplementary Table S4). These drugs had been stopped at least 12 months before venesection, except in three patients who had ceased interferon-β therapy 6 months before. Disability at the time of venesection was assessed using the Kurtzke Expanded Disability Status Scale (EDSS),^82^ and the MS Severity Score was determined from the EDSS and disease duration.^83^ There were no significant differences in age (*P*=0.670) or sex (*P*=0.162) between the healthy subjects and the total group of MS patients.

### Processing of blood samples

Ten ml of blood was used for DNA extraction and HLA typing, and five ml for serum collection. PBMC were separated by density centrifugation, resuspended in complete RPMI with 10% dimethyl sulphoxide (Sigma, St Louis, MO, USA) and cryopreserved in liquid nitrogen, as previously described.^19^

### Anti-viral antibody assays

IgG seroreactivity to EBV and CMV was assessed using ELISA kits (MP Biomedicals, Santa Ana, CA, USA) to detect IgG specific for EBNA1, VCA and CMV. In EBV-seropositive subjects we also used ELISA kits (MP Biomedicals) to determine IgM and IgA reactivity to VCA, and IgG, IgM and IgA reactivity to EA. To determine the titres of anti-EBNA1 IgG and anti-VCA IgG we diluted the serum samples in doubling dilutions ranging from 1/50 to 1/12 800 before testing by ELISA, as previously described.^19^

### HLA typing

Genomic DNA was extracted from 10 ml heparinized blood using NucleoSpin Blood XL DNA extraction kits (Macherey-Nagel, Düren, Germany). HLA-A and HLA-B typing was performed by Pathology Queensland (Royal Brisbane and Women’s Hospital, Brisbane, Queensland, Australia) using commercially available sequence-specific oligonucleotide probes bound to colour-coded microbeads (Luminex technology, Luminex Corporation, Austin, TX, USA). We performed HLA-class I typing on 50 of the healthy controls and 59 of the patients with MS (Supplementary Table S5).

### Generation of LCL

EBV-infected LCL were generated from each subject by incubating 3–5 × 10^6^ washed PBMC with the B95-8 strain of EBV overnight in 1 ml RPMI-C supplemented with 2 μg ml^−1^ cyclosporin A (Sigma), as previously described.^19^ LCL were cultured in this medium for a month followed by at least another 2 months of culture in RPMI-C without cyclosporin A until confluent, rapidly growing lines were obtained. Each was grown for the final week of culture in complete phenol-red-free RPMI and checked by flow cytometry to ensure there was no evidence of T-cell contamination, as determined by CD3 expression or intracellular IFN-γ expression, before being used in T-cell assays. To determine the frequency of lytically infected cells in LCL we used flow cytometry to measure expression of the EBV immediate early protein BZLF1 with the BZ1 monoclonal antibody (Santa Cruz Biotechnology, Dallas, TX, USA) in LCL of 19 healthy EBV-seropositive subjects and 56 patients with MS at the same time as the measurement of the LCL-specific T-cell frequency.

### Flow cytometry and intracellular cytokine staining

Cryopreserved PBMC samples were thawed and cultured for 24 h before use to allow cells to rest and re-express cell surface receptors. Stained PBMC samples were assessed using a Beckman Coulter Gallios flow cytometer with 10 colour acquisition capability (Beckman Coulter, Brea, CA, USA). For all flow cytometry cellular acquisition panels, 400 000 events were collected to enable accurate estimation of antigen-specific T-cell frequencies. Single-labelled tubes for each antibody, isotype-matched control antibodies, fluorescence-minus-one controls, dead cell exclusion dyes and doublet discrimination were used extensively during panel development to ensure accurate positive cutoff values and compensation matrices and to validate cell phenotype detection sensitivity and resolution. Flow cytometry data was analysed using Kaluza 1.3 software (Beckman Coulter).

Firstly, in 95 patients with MS and in 56 healthy subjects, we employed intracellular cytokine staining to measure the frequency of T cells producing IFN-γ in response to stimulation with autologous LCL, a pool of 13 HLA-class-I-restricted peptides from EBV latent proteins, a pool of five HLA-class-I-restricted peptides from EBV lytic proteins and a pool of 18 HLA-class-I-restricted peptides from CMV (Table 2). These peptides were selected from published lists of EBV peptides^8^ and CMV peptides^84, 85^ on the basis of their being restricted by HLA-A or HLA-B molecules frequently carried by MS patients and healthy subjects, namely HLA-A*01, HLA-A*02, HLA-A*03, HLA-A*11, HLA-B*07, HLA-B*08, HLA-B*35 and HLA-B*44, as we have previously reported^19^ and as shown for the subjects in this study in Supplementary Table S5. The peptides were synthesized by Auspep Pty Ltd (Tullamarine, Victoria, Australia) at ⩾95% purity. Purity was assessed by high-performance liquid chromatography, and product molecular weight confirmed by mass spectral analysis. For antigenic stimulation of PBMC and subsequent assessment of intracellular IFN-γ expression, cultures of 10^6^ PBMC were mixed with either 5 × 10^5^ autologous LCL, the EBV lytic peptide pool, the EBV latent peptide pool or the CMV peptide pool (each peptide at 2 μg ml^−1^). Non-stimulated PBMC cultures were used to measure background IFN-γ expression. As a positive control, cells were stimulated with the T-cell mitogens, phytohaemagglutinin or phorbol myristate acetate/ionomycin C (Becton Dickinson, Franklin Lakes, NJ, USA). Cultures were stimulated for 5 h in the presence of Brefeldin A (Becton Dickinson) and washed before staining with Aqua live/dead cell exclusion dye (Invitrogen, Carlsbad, CA, USA). After washing, cells were stained with antibodies to cell surface markers for 30 min at 4 °C and then fixed and permeabilized before intracellular IFN-γ staining. Samples were run on the flow cytometer within 12 h of completion of cell staining.

We used the following fluorochrome-conjugated antibody panel: anti-IFN-γ-FITC (Beckman Coulter) (FL1), anti-PD-1(CD279)-PE (Becton Dickinson)/Tim-3 PE (Biolegend, San Diego, CA, USA) (FL2), anti-CCR7-PerCP-Cy5.5 (Becton Dickinson) (FL4), anti-CD45RA-PE-Cy7 (Becton Dickinson) (FL5), anti-CD3-APC (Beckman Coulter) (FL6), anti-CD8-APC-A700 (Beckman Coulter) (FL7), anti-CD62L-APC-Cy7 (Biolegend) (FL8), anti-CD4-V450 (Becton Dickinson) (FL9) and Aqua Live/Dead cell exclusion dye (Invitrogen) (FL10). This panel was designed to measure the proportions of T cells producing IFN-γ in response to test antigens within the CD4^+^ population, the CD8^+^ population, the naive (CD3^+^CD4/8^+^CD45RA^+^CD62L^+^), central memory (CD3^+^CD4/8^+^CD45RA^−^CD62L^+^), effector memory (CD3^+^CD4/8^+^CD45RA^−^CD62L^−^) and EMRA (CD3^+^CD4/8^+^CD45RA^+^CD62L^−^) subsets, and also within PBMC as a whole. To analyse these T-cell subsets we used the same gating strategy as previously depicted.^13^ T-cell exhaustion markers Tim-3 and PD-1 were used to assess T-cell exhaustion. We calculated the proportion of CD4^+^ or CD8^+^ T cells of each memory phenotype within PBMC, and from this determined the frequency of antigen-specific T cells within each cellular phenotype and within PBMC. We calculated the number of antigen-specific IFN-γ-expressing cells by subtracting the number of IFN-γ-expressing cells in the absence of antigenic stimulation (background) from the number of IFN-γ-expressing cells in the presence of antigenic stimulation.

### Assessment of T-cell polyfunctionality

To assess T-cell polyfunctionality, we used flow cytometry and intracellular cytokine staining to measure the frequencies of CD8^+^ T cells producing IL-2, TNF-α and IFN-γ in response to stimulation with selected HLA-A*02-restricted peptides and to measure the frequencies of T cells binding to the corresponding HLA-peptide complexes stabilized on a dextran polymer backbone with attached fluorophores (Dextramers) in 20 HLA-A*02^+^ healthy subjects and 20 HLA-A*02^+^ MS patients. Because peptide stimulation greatly reduced Dextramer binding within a few hours by downregulating the T-cell receptor, it was necessary to stain one separate tube of unstimulated cells with an eight colour Dextramer/phenotype flow cytometry panel to measure the frequencies of T cells binding to the Dextramers, and to use another tube of either peptide-stimulated (5 h) or unstimulated cells stained with an eight colour cytokine/phenotype flow cytometry panel to assess cytokine production. We used the following HLA-A*02-restricted peptides: LLDFVRFMGV derived from the EBV latent protein EBNA3C, CLGGLLTMV and FLYALALL from the EBV latent protein LMP2A, and GLCTLVAML from the EBV lytic protein BMLF1, and obtained the corresponding HLA-A*02-peptide Dextramers from Immudex, Copenhagen, Denmark.

Unstimulated cells were stained with the eight colour Dextramer/phenotype flow cytometry panel as follows: Aqua viability staining (FL10), followed by surface staining with each PE-conjugated Dextramer (FL2) for 15 min before the addition of anti-CD57-FITC (Becton Dickinson) (FL1), anti-CD8-PE-CF594 (Becton Dickinson) (FL3), anti-CD45RA-PE-Cy7 (FL5), anti-CD27-APC (Becton Dickinson) (FL6), anti-CD62L-APC-Cy7 (FL8) and anti-PD-1(CD279)-BV421 (Becton Dickinson) (FL9). After stimulation with each peptide, or in the absence of peptide, for 5 h in the presence of Brefeldin A, monensin (Becton Dickinson) and anti-CD107a-PE (Becton Dickinson) (FL2), PBMC were stained with the eight colour cytokine/phenotype flow cytometry panel as follows: Aqua viability staining (FL10) followed by surface staining with anti-CD8-PE-CF594 (FL3), anti-CD45RA-PE-Cy7 (FL5), anti-CD62L-APC-Cy7 (FL8), followed by fixation/permeabilization and intracellular staining with anti-IFN-γ-FITC (Becton Dickinson) (FL1), anti-IL-2-APC (Becton Dickinson) (FL6) and anti-TNF-α-BV421 (Becton Dickinson) (FL9). We calculated the number of peptide-specific cytokine-producing cells by subtracting the number of cytokine-producing cells in the absence of peptide stimulation (background) from the number of cytokine-producing cells in the presence of peptide stimulation. To quantify T-cell polyfunctionality, we calculated the polyfunctionality index^35^ which gives successively higher weightings to the frequencies of cells producing one, two and three cytokines; we then divided this index by the frequency of T cells binding the respective HLA-peptide Dextramer. The formula for the polyfunctionality index was: (0/3 × frequency of cells within the CD8^+^ population producing no cytokines)+(1/3 × frequency of cells within the CD8^+^ population producing one cytokine)+(2/3 × frequency of cells within the CD8^+^ population producing two cytokines)+(3/3 × frequency of cells within the CD8^+^ population producing three cytokines). For the construction of pie charts, we calculated the frequency of Dextramer^+^ cells not secreting any cytokines by subtracting frequencies of peptide-stimulated cytokine-producing cells from the total frequency of Dextramer^+^ cells.

### Quantification of EBV genome load

Total DNA was extracted from PBMC using the QIAmp DNA blood mini kit (Qiagen, Hilden, Germany) then subjected to a real-time PCR assay on the Rotorgene 3000 (Qiagen). DNA from the Namalwa cell line (two copies of EBV per cell) was used to set up a standard curve to determine the unknown EBV copies of the samples in the assay. The primers and probe were directed towards a conserved portion of the *BamH1W* segment of the EBV genome.^36^

### Statistical analysis

Statistical analyses were performed using GraphPad Prism version 7.00 (Graphpad Software Inc, San Diego, CA, USA) and Sigmaplot 12.5 (Systat Software Inc, Chicago, IL, USA). Because most of the data was not normally distributed, the results were presented as medians with interquartile ranges. For single comparisons between the whole group of MS patients and healthy subjects or between patients during clinical attacks and patients not having an attack, Student’s *t-*test or the Mann–Whitney rank sum test was used, according to the distribution of the data, as determined by the D’Agostino–Pearson omnibus normality test (GraphPad Prism). To assess the relationships between T-cell frequencies and age, disease duration, EDSS score, Multiple Sclerosis Severity Score, EBV genome load and anti-EBV antibody titres we used Spearman rank correlation because the data was not normally distributed. For analysis of the proportions of healthy subjects and patients producing anti-EBV antibodies we employed *χ*^2^, *χ*^2^ with Yates correction or Fisher’s exact test according to Prism usage recommendations. Differences were considered significant for *P*<0.05.

## Figures and Tables

**Table 1:** General characteristics of healthy subjects and patients with MS

	*HC (*n*=56)*	*All MS (*n*=95)*	*CIS (*n*=8)*	*RRMS (*n*=33)*	*SPMS (*n*=31)*	*PPMS (*n*=23)*
Age (years)	44.0 (32.3–52.8)	43.0 (37.0–54.0)	38.5 (26.3–42.5)	39.0 (33.0–47.0)	48.0 (41.0–56.0)	51.0 (39.0–56.0)
Sex (% female)	83.9	72.6	50.0	84.8	74.2	60.8
Age of onset of MS (years)		34.0 (26.0–40.0)	36.3 (25.0–41.9)	32.0 (27.0–37.8)	28.0 (24.0–39.0)	37.0 (33.0–48.0)
Duration of MS (years)		8.0 (3.0–17.0)	0.5 (0.1–4.1)	3.0 (1.0–10.0)	16.0 (10.0–25.0)	9.0 (5.0–18.0)
EDSS score		5.0 (2.5–6.5)	0.0 (0.0–2.5)	2.0 (1.0–3.5)	6.5 (5.5–8.0)	6.5 (5.5–8.0)
MSSS		6.6 (3.7–8.8)	0.7 (0.4–6.6)	4.6 (2.4–6.6)	7.5 (5.6–9.1)	8.3 (6.6–9.7)

Abbreviations: All MS, total group of MS patients; CIS, clinically isolated syndrome; EDSS, Expanded Disability Status Scale; HC, healthy control subjects; MS, multiple sclerosis; MSSS, MS Severity Score; SPMS, secondary progressive MS; PPMS, primary progressive MS; RRMS, relapsing–remitting MS.

All values except for sex presented as medians (interquartile ranges).

**Table 2:** EBV and CMV peptides

*EBV*				*CMV*			
*Protein*	*Epitope sequence*	*HLA restriction*	*Epitope code*	*Protein*	*Epitope sequence*	*HLA restriction*	*Epitope code*
**EBNA1**	HPVGEADYFEY	B35	HPV	pp150 (UL32)	TTVYPPSSTAK	A3	TTV
**EBNA3A**	RLRAEAQVK	A3	RLR	pp50 (UL44)	VTEHDTLLY	A1	VTE
**EBNA3A**	RPPIFIRRL	B7	RPP	gB (UL55)	IMREFNSYK	A3	IMR
**EBNA3A**	FLRGRAYGL	B8	FLR	pp65 (UL83)	YSEHPTFTSQY	A1	YSE
**EBNA3A**	YPLHEQHGM	B35	YPL	pp65 (UL83)	NLVPMVATV	A2	NLV
**EBNA3B**	AVFDRKSDAK	A11	AVF	pp65 (UL83)	ATVQGQNLK	A11	ATV
**EBNA3B**	IVTDFSVIK	A11	IVT	pp65 (UL83)	GPISGHVLK	A11	GPI
**EBNA3B**	AVLLHEESM	B35	AVL	pp65 (UL83)	RPHERNGFTV	B7	RPH
**EBNA3B**	VEITPYKPTW	B44	VEI	pp65 (UL83)	TPRVTGGGAM	B7	TPR
**EBNA3C**	LLDFVRFMGV	A2	LLD	pp65 (UL83)	FPTKDVAL	B35	FPT
**EBNA3C**	QPRAPIRPI	B7	QPR	pp65 (UL83)	IPSINVHHY	B35	IPS
**EBNA3C**	EENLLDFVRF	B44	EEN	pp65 (UL83)	QEFFWDANDI	B44	QEF
**LMP2A**	CLGGLLTMV	A2	CLG	pp65 (UL83)	SEHPTFTSQY	B44	SEH
BRLF1	YVLDHLIVV	A2	YVL	IE-1 (UL123)	VLAELVKQI	A2	VLA
BRLF1	ATIGTAMYK	A11	ATI	IE-1 (UL123)	VLEETSVML	A2	VLE
BMLF1	GLCTLVAML	A2	GLC	IE-1 (UL123)	YILEETSVM	A2	YIL
BZLF1	RAKFKQLL	B8	RAK	IE-1 (UL123)	ELRRKMMYM	B8	ELR
BZLF1	EPLPQGQLTAY	B35	EPL	IE-1 (UL123)	QIKVRVDMV	B8	QIK

Abbreviations: CMV, cytomegalovirus; EBV, Epstein–Barr virus; HLA, human leukocyte antigen.

For each protein, the peptides are listed in alphabetical and numerical order of the HLA restricting allele. For peptides of a given protein restricted by the same allele, the peptides are listed alphabetically according to amino acid sequence. The epitope code consists of the first three letters of the sequence. EBV latent proteins are highlighted in bold; all other EBV and CMV proteins are from the lytic phase of infection. These peptides were selected from published lists of EBV peptides^8^ and CMV peptides.^84, 85^
