# Defective T-cell control of Epstein–Barr virus infection in multiple sclerosis

**DOI:** 10.1038/cti.2017.25

**Published:** 2017-06-16

**Authors:** Michael P Pender, Peter A Csurhes, Jacqueline M Burrows, Scott R Burrows

**Correction to:** *Clinical & Translational Immunology* (2017) **6**, e126; doi:10.1038/cti.2016.87; published online 20 January 2017.

The volume number in the PDF of the above paper was published incorrectly. It should be 6 instead of 5. This has now been corrected in the PDF.

The publishers wish to apologize for any inconvenience caused.

