# Supplementary material for: Defective T-cell control of Epstein–Barr virus infection in multiple sclerosis
Source: Clin Transl Immunology. 2017 Jan 20;6(1):e126–. doi: 10.1038/cti.2016.87 (PMC5292561; doi:10.1038/cti.2016.87)
Supplement: Supplementary Information [file cti201687x1.pdf]

Supplementary Information

Table S1 Peripheral blood T cell frequencies

	$CD3^+$	$CD4^+CD3^+$	$CD8^+CD3^+$	CD4:CD8 ratio
HC (*n* = 56)	70.88 (64.59–74.21)	44.74 (38.96–50.97)	18.69 (16.05–23.56)	2.25 (1.75–3.14)
MS (*n* = 95)	66.73 (58.36–72.38)	45.29 (38.87–51.39)	15.57 (12.63–20.40)	2.94 (2.12–3.64)
P value	0.006[a]	0.845[b]	0.001[b]	0.006[a]

Median (interquartile range) percentages of T cells within PBMC. HC: healthy control subjects. [a]Mann–Whitney rank sum test; [b]Student's *t* test. This data is derived from the results of our previous study on memory T cell subsets,[13] which included all of the subjects in the present study as well as additional ones.

Table S2 Frequencies of memory T cell subsets

	CD4$^+$				CD8$^+$				
	N	CM	EM	EMRA	N	CM	EM	EMRA	EM/EMRA
HC (*n* = 56)	10.29 (7.16–16.54)	8.99 (5.69–12.34)	14.44 (11.30–19.76)	7.29 (4.28–8.92)	3.99 (2.33–5.22)	1.50 (0.99–2.17)	6.76 (4.53–8.07)	6.11 (4.66–9.36)	13.00 (10.53–16.52)
MS (*n* = 95)	9.63 (5.65–13.54)	7.45 (4.63–10.64)	16.77 (11.4–22.94)	7.08 (4.55–11.69)	2.88 (1.73–4.63)	1.53 (0.84–2.23)	5.31 (3.43–6.68)	5.06 (3.43–7.34)	10.60 (7.69–14.06)
P value[a]	0.130	0.048	0.165	0.443	0.083	0.870	0.002	0.015	0.001

Median (interquartile range) percentages of memory T cell subsets within PBMC. N: naïve T cells; HC: healthy control subjects. [a]Mann–Whitney rank sum test. This data is derived from the results of our previous study on memory T cell subsets,[13] which included all of the subjects in the present study as well as additional ones.

Table S3 Frequencies of LCL-specific T cells in PBMC in EBV-seronegative and EBV-seropositive healthy subjects

	CD3⁺	CD4⁺	CD8⁺	CD8⁺ N	CD8⁺ CM	CD8⁺ EM	CD8⁺ EMRA
EBV-seronegative (*n* = 6)	0.000 (0.000–0.001)	0.000 (0.000–0.002)	0.000 (0.000–0.001)	0.000 (0.000–0.000)	0.000 (0.000–0.000)	0.001 (0.000–0.001)	0.000 (0.000–0.000)
EBV-seropositive (*n* = 56)	0.283 (0.136–0.395)	0.035 (0.016–0.073)	0.183 (0.089–0.304)	0.000 (0.0–0.001)	0.003 (0.002–0.008)	0.152 (0.071–0.261)	0.013 (0.004–0.056)
P value[a]	< 0.0001	< 0.0001	< 0.0001	0.018	< 0.0001	< 0.0001	< 0.0001

Median (interquartile range) percentages of LCL-specific T cells within PBMC. N: naïve T cells. [a]Mann–Whitney rank sum test.

Table S4 Immunomodulatory therapies received by MS patients prior to venesection

	Number of patients treated	Percentage of patients treated[a]	Median time between treatment cessation and venesection (years)
Any[b]	20	21.1	
Interferon-β	16	16.8	3
Glatiramer acetate	5	5.3	5
Methotrexate	3	3.2	1
Azathioprine	2	2.1	7
Natalizumab	1	1.1	1
Sulphasalazine[c]	1	1.1	2

[a]Percentage of treated patients within the total group of 95 MS patients. [b]Some patients had been treated with more than one immunomodulatory therapy prior to venection. [c]For polyarthropathy.

Table S5 HLA class I typing in healthy subjects and MS patients

HLA-A Allele	HC n (%)[a]	MS n (%)[a]	HLA-B Allele	HC n (%)[a]	MS n (%)[a]
A*01	19 (37.3)	19 (32.2)	B*07	14 (28.0)	21 (35.6)
A*02	29 (56.9)	21 (35.6)	B*08	12 (24.0)	8 (13.6)
A*03	9 (17.6)	17 (28.8)	B*13	4 (8.0)	1 (1.7)
A*11	4 (7.8)	7 (11.9)	B*14	8 (16.0)	6 (10.2)
A*23	2 (3.9)	2 (3.4)	B*15	6 (12.0)	8 (13.6)
A*24	4 (7.8)	13 (22.0)	B*18	2 (4.0)	8 (13.6)
A*25	3 (5.9)	5 (8.5)	B*27	3 (6.0)	5 (8.5)
A*26	0 (0.0)	1 (1.7)	B*35	7 (14.0)	10 (16.9)
A*28	2 (3.9)	0 (0.0)	B*37	1 (2.0)	3 (5.1)
A*29	5 (9.8)	8 (13.6)	B*38	3 (6.0)	0 (0.0)
A*30	2 (3.9)	3 (5.9)	B*39	1 (2.0)	6 (10.2)
A*31	3 (5.9)	3 (5.1)	B*40	6 (12.0)	3 (5.1)
A*32	5 (9.8)	4(6.8)	B*41	0 (0.0)	2 (3.4)
A*33	0 (0.0)	4 (6.8)	B*44	11 (22.0)	18 (30.5)
A*34	0 (0.0)	1 (1.7)	B*45	0 (0.0)	2 (3.4)
A*36	0 (0.0)	1 (1.7)	B*46	0 (0.0)	1 (1.7)
A*43	0 (0.0)	0 (0.0)	B*49	3 (6.0)	1 (1.7)
A*66	0 (0.0)	0 (0.0)	B*50	0 (0.0)	0 (0.0)
A*68	4 (7.8)	4 (6.8)	B*51	3 (6.0)	4 (6.8)
A*69	0 (0.0)	0 (0.0)	B*52	0 (0.0)	1 (1.7)
A*74	0 (0.0)	0 (0.0)	B*53	0 (0.0)	0 (0.0)
A*92	0 (0.0)	0 (0.0)	B*55	2 (4.0)	2 (3.4)
			B*56	1 (2.0)	1 (1.7)
			B*57	3 (6.0)	3 (5.1)
			B*58	1 (2.0)	0 (0.0)
			B*59	0 (0.0)	0 (0.0)
			B*60	1 (2.0)	0 (0.0)
			B*62	1 (2.0)	0 (0.0)
			B*78	0 (0.0)	0 (0.0)

HLA-A and HLA-B typing results in 50 healthy control subjects (HC) and 59 MS patients. [a]Number (%) of subjects carrying each allele, either heterozygously or homozygously.
